# Immunological Considerations for *Schistosoma* Vaccine Development: Transitioning to Endemic Settings

**DOI:** 10.3389/fimmu.2021.635985

**Published:** 2021-03-04

**Authors:** Emmanuella Driciru, Jan Pieter R. Koopman, Stephen Cose, Afzal A. Siddiqui, Maria Yazdanbakhsh, Alison M. Elliott, Meta Roestenberg

**Affiliations:** ^1^Immunomodulation and Vaccines Programme, Medical Research Council/Uganda Virus Research Institute and London School of Hygiene & Tropical Medicine Uganda Research Unit, Entebbe, Uganda; ^2^Department of Parasitology, Leiden University Medical Center, Leiden, Netherlands; ^3^Center for Tropical Medicine and Infectious Diseases, Texas Tech University School of Medicine, Lubbock, TX, United States; ^4^Department of Internal Medicine, Center for Tropical Medicine and Infectious Diseases, Texas Tech University Health Sciences Center, Lubbock, TX, United States

**Keywords:** schistosomiasis, vaccine-candidate, praziquantel, co-infections, endemic, vaccine response, co-administration, co-delivery

## Abstract

Despite mass drug administration programmes with praziquantel, the prevalence of schistosomiasis remains high. A vaccine is urgently needed to control transmission of this debilitating disease. As some promising schistosomiasis vaccine candidates are moving through pre-clinical and clinical testing, we review the immunological challenges that these vaccine candidates may encounter in transitioning through the clinical trial phases in endemic settings. Prior exposure of the target population to schistosomes and other infections may impact vaccine response and efficacy and therefore requires considerable attention. Schistosomes are known for their potential to induce T-reg/IL-10 mediated immune suppression in populations which are chronically infected. Moreover, endemicity of schistosomiasis is focal whereby target and trial populations may exhibit several degrees of prior exposure as well as *in utero* exposure which may increase heterogeneity of vaccine responses. The age dependent distribution of exposure and development of acquired immunity, and general differences in the baseline immunological profile, adds to the complexity of selecting suitable trial populations. Similarly, prior or concurrent infections with other parasitic helminths, viral and bacterial infections, may alter immunological responses. Consequently, treatment of co-infections may benefit the immunogenicity of vaccines and may be considered despite logistical challenges. On the other hand, viral infections leave a life-long immunological imprint on the human host. Screening for serostatus may be needed to facilitate interpretation of vaccine responses. Co-delivery of schistosome vaccines with PZQ is attractive from a perspective of implementation but may complicate the immunogenicity of schistosomiasis vaccines. Several studies have reported PZQ treatment to induce both transient and long-term immuno-modulatory effects as a result of tegument destruction, worm killing and subsequent exposure of worm antigens to the host immune system. These in turn may augment or antagonize vaccine immunogenicity. Understanding the complex immunological interactions between vaccine, co-infections or prior exposure is essential in early stages of clinical development to facilitate phase 3 clinical trial design and implementation policies. Besides well-designed studies in different target populations using schistosome candidate vaccines or other vaccines as models, controlled human infections could also help identify markers of immune protection in populations with different disease and immunological backgrounds.

## Introduction

Schistosomiasis is a poverty associated chronic disease caused by parasitic trematodes of the *genus Schistosoma* (1). Over 190 million people globally are actively infected, of which 90% live in Africa (2) and over 200,000 deaths result from schistosomiasis disease annually in Sub-Saharan Africa (SSA) (1). The two main forms of African schistosomiasis, caused by *Schistosoma haematobium* and *S. mansoni* parasites, affect the urinary and gastro-intestinal tract, respectively. The intermediate host, fresh water snails of the genus *Bulinus* and *Biomphalaria* shed infective cercariae in water where they penetrate the skin of the human host. Water-related livelihood activities, such as fishing, thus drive transmission in resource limited settings with poor hygiene and sanitary facilities (3). Following exposure, schistosomula migrate through the blood stream and lungs to the mesenteric and/or peri-vesical vessels intestinal venules where they mature into female and male adult worms. Adult worms mate and the female produces eggs that are released from the intestinal or urinary tract to complete the cycle. Pathology results mainly from accumulation of deposited eggs that induce inflammatory responses, granuloma formation and fibrosis. This results in strictures and calcification in the urinary tract due to *S. haematobium* egg deposition, and liver fibrosis from *S. mansoni* egg deposition in the Liver. Long term consequences include hydronephrosis and kidney failure in the case of *S. haematobium* infection, and hepatosplenomegaly and portal hypertension in the case of *S. mansoni* infection, accounting for the morbidity and mortality in schistosomiasis disease (4, 5).

In the last decade, the World Health Organization (WHO) set an ambitious goal of controlling schistosomiasis by the year 2020 and eliminating it as a public health burden by 2025. Initially, increasing coverage of mass drug administration (MDA) of praziquantel (PZQ), the only drug currently used for schistosomiasis treatment (6), was thought to be sufficient to achieve this. Unfortunately, the increased coverage of MDA decreases the intensity of infection and thus suffices as a tool to alleviate schistosomiasis morbidity but does not prevent re-infection. Consequently, prevalence can be restored in 6–8 months after PZQ administration (7). As a result, control programs rely on the repeated administration of PZQ, whilst facing significant challenges with drug uptake, adherence and sustainability. Hence, schistosomiasis remains a paramount public health concern and economic burden in the resource limited countries (6). Also, the full dependency on a single drug poses a threat of drug resistance (7). This leaves vaccination a key approach for the control and possible elimination of schistosomiasis. An effective vaccine could contribute to prevention and protection against re-infection. Consequently, schistosomiasis has been ranked among the top ten diseases for which a vaccine is urgently needed (8). The current overall consensus on preferred product characteristics (PPC) for an effective prophylactic vaccine is induction of 75% reduction in worm burden in immunized individuals and egg excretion in infected patients (9).

However, development of a novel schistosome vaccine faces several scientific challenges due to the immune evasive nature of adult schistosomes and schistosome-induced host immune-modulation. Illustrated by the fact that no human anti-helminth vaccines exists, the development of a schistosomiasis vaccine is likely to be more complex as compared to the recent rapid development of SARS-CoV-2 vaccines. Most importantly, the multi-stage nature of schistosomes, transforming from cercariae to Schistosomula, adult worms and ultimately eggs, involves complex antigenic switches which are life-stage specific, but can also be present across life stages. In addition, the induction of IgE responses, associated risk of allergic reactions and the potential of aggravating granulomas and fibrosis by egg-mediated responses (10) makes schistosomiasis vaccine development complex.

Moreover, poly-parasitism and co-infections, a predominant phenomenon in schistosomiasis endemic regions, mediate cross-immune-regulation whereby one infection influences susceptibility, intensity and immune responses to the other (11). In addition, there is inadequate understanding of how past and existing schistosome infections, repeated exposure, poly-parasitism and prior treatment explicitly structure the immune system in individuals, within and between the different populations. These factors are likely to increase heterogeneity in vaccine responses. Further, the need to implement vaccines in the context of the current MDA control programmes necessitates an integrated approach to praziquantel and vaccine administration (9). However, co-administration of vaccines and praziquantel presents divergent immunological dynamics that have to be considered. Here, we review the immunological challenges for schistosome vaccine development and testing in endemic settings and provide perspectives of how this scientific priority area can be accelerated.

### The Progress Toward a Schistosome Vaccine

The initial approach in the quest for a schistosomiasis vaccine involved vaccinating mice with live attenuated cercariae and schistosomula followed by a challenge infection to assess protectivity. These studies demonstrated that 60–70% protection was achievable with a single immunization and could be boosted up to over 90% with subsequent immunisations (12, 13). This approach was similarly efficacious in baboons eliciting protection of over 50% to schistosome larvae challenge and 89% reduction in worm burden, upon immunization with gamma-irradiated cercariae (14). However, despite induction of close to 100% protection (13), this approach is difficult to translate to humans (15).

Nevertheless, these studies provided a strong indication for schistosome vaccine discovery, and knowledge on induction of protective immunity. Acquired immunity was found to be mainly associated with a Th1 type IFN-γ and TNFα pulmonary inflammatory and antibody response, IL-4, IL-5, and eosinophils are of negligible importance meanwhile, IL-10 and IgG4 negates protection and are associated with re-infection (9, 13, 14). Conclusively, these studies denoted that excessive induction of either Th2 or Th1 could lead to damaging pathology and thus, induction a balanced Th1 and Th2 response is fundamental for achieving optimal protection (15).

Utilizing advancements in technology, vaccine development has, in the recent past, shifted focus from irradiated parasite vaccines to specific antigen molecules. Nonetheless, vaccination with irradiated *S. mansoni* cercarie has contributed to the identification of five such molecules including the Sh28GST and the Sm14 which have proceeded to human clinical trials (12, 15) as discussed below and further reviewed by Molehin (9) and McManus et al. (15)

#### *S. haematobium* 28-kD Glutathione S-Transferase (r*Sh*28GST)

This glutathione S-transferase (GST) antigen is a worm detoxification enzyme which is important in preventing parasite oxidative stress (16). Ultrastructural localization of the 28GST show a dense cytosolic, as well as genital, tegumental surface and parenchyma distribution in the schistosome parasite (17). Thus, the 28GST is closely associated with the parasites' muscular organs and anti-28GST specific antibodies mediate Antibody-Dependent-Cellular-Cytoxicity (ADCC) and also inhibit transferase activity of the enzyme interfering with muscle function and ultimately in reduced worm fecundity (18, 19).

Rodent studies demonstrate that 28-kDa fraction of GST (*Sh*28GSGT) elicits an antibody response which is capable of inhibiting the native GST enzymes, conferring between 40 and 70% protection in rats, mice, hamsters and baboons (20). Experimental infection studies in non-human primate models revealed an anti-fecundity effect with significant reduction in tissue egg load and fecal egg excretion despite a lack of effect on adult worm burden (21).

The phase I trial reported safety and tolerability of the r28GST in Alum formulation in human subjects. The immunological read-abouts demonstrated that 28GST vaccine candidate is highly immunogenic, inducing high levels of specific IgG1, IgG2, IgG3 antibodies. A strong Th2 cytokine response was also observed, typically IL-5 and IL-13 cytokines (22). Though findings from the phase II trial are yet to be published, the phase III trial, a randomized parallel-group double-blinded trial in school children in endemic Senegal, reported a good tolerance of the vaccine with induction of long lasting vaccine specific response. High titres of IgG1, IgG2, and IgG were induced in the vaccine group. However, this vaccine candidate did not show sufficient protection and this was attributed to failure to induce the desired specific-IgG3. The investigators of this trial also attribute this to interreference by the praziquantel treatment that was administered after the first vaccine dose and before the booster dose (18). Post vaccination treatment with PZQ, which coincides with elaboration of immune response to a vaccine, has been shown to interfere with cytokine response (18, 23, 24).

#### *S. mansoni* 14-kDA Fatty Acid Binding Protein (*Sm*14)

Sm14 is one of the fatty acid binding (FABP) proteins that play a crucial role in the uptake, transport and compartmentalization of host-derived sterols by schistosomes (25). Sm-14 protein enzymes are localized in the cytosol and in tissues adjoining the interfaces of parasite/host contact such as the basal lamella of the worm tegument and muscles layers to enable acquisition of lipids from the host (26). They are also found in the gut epithelium for lipid transportation and utilization throughout the parasite. Sm14-specific antibodies bind to tubercles on the parasite's dorsal surface and interfere with uptake of lipids essential for parasite survival as well as mediate ADCC (27).

In rodent studies, Vaccination with the rSm14 induced up to 66 and 89% protection, of Swiss mice and New Zealand white rabbits with, respectively (28). Sera from immunized mice showed generally high reactivity and significant level of rSm14-specific IgM, although IgG and IgA titres were low (29).

In the phase I trial, the *Sm*14/GLA-SE formulation was safe, highly tolerable and immunogenic in adult male human subjects. It induced a strong CD4+ T cell response producing single Th1 cytokines, particularly the TNF and IL-2. Also, high titres of Sm14-specific IgG, IgG1, and IgG3 antibodies were elicited in vaccinated individuals (27, 30). The *Sm*14 vaccine has now advanced to a phase IIb trial to be conducted in school children in the endemic Senegal river basin region (25) (NCT03799510).

#### *S. mansoni* Tetraspanin-2 (*Sm*-TSP-2)

*Sm*-tetraspanin is a member of a four-domain-structured tetraspanin surface membrane protein linked by two extracellular loops and made of two types, TSP-1 and TSP2 (31). The main vaccine antigen in the Sm-TSP candidate vaccine is comprised of the extracellular loop and the TSP-2 type. The TSP-2 antigen was found to be more strongly recognized by IgG1 and IgG3 antibodies in the sera of naturally immune populations, unlike TSP-1 (32). The TSP-2 is a readily immune-accessible antigen on the surface of newly transformed schistosomula and a critical tegument protein for nutrient acquisition, waste excretion and immune evasion. Anti-TSP-2 antibodies interfere with these parasite survival mechanisms and elicit a protective immunity against infection in a vaccinated host (32).

Immunization with *Sm*-TSP-2 result in 57% and 62% reduction in worm and liver egg burden, respectively, and 69% reduction in fecal egg count, in rodent models (32). An increased production of Sm-TSP-2 specific antibodies and IL-4, IL-10 cytokines by spleen cells is also observed in immunized animals (33).

Phase Ia and phase Ib trials of the Sm-TSP-2/Alhydrogel (Sm-TSP-2/AI) have both been initiated to investigate safety and immunogenicity of this vaccine candidate in human subject (NCT03910972). Phase I trial of the Sm-TSP/Alhydrogel with or without glucopyranosyl lipid A (GLA-AF) formulation in non-endemic setting show the vaccine is safe and tolerable among *Sm*-naïve individuals (34). The “Sm-TSP/Al with GLA-AF” formulation elicited higher sero-response than the “Sm-TSP/Al without GLA-AF” and placebo, with a dose-response relationship exhibited by Sm-TSP/Al with GLA-AF (34).

#### *S. mansoni Sm*-p80/GLA-SE

*Sm*-p80 is the large subunit of calpain, a calcium activated neutral tegument protease, which is located in and on the surface epithelial syncytium and mediates tegument biogenesis for host immune evasion. *Sm*-p80 is a highly immuno-dominant membrane antigen with no cross-reactivity with vertebrate calpains (35).

A multitude of studies performed *in vitro*, in rodents and non-human primates over the past 23 years have demonstrated that *Sm*-p80 is a very promising vaccine candidate with prophylactic, therapeutic, anti-pathology and transmission blocking efficacies (36). Significant reduction in adult worm burden, tissue egg load and fecal egg excretion following *Sm*-p80 vaccination has been demonstrated, whereas potentially allergic IgE responses have not been registered (37). *Sm*-p80 immunization elicits significant complement-dependent killing of schistosomula (36). Administration of PZQ preceding *Sm*-p80 vaccination is proven to profoundly reduce tissue egg retention and hatching in non-human primates (38, 39). Following the desirable responses and results from rodent and non-human primate studies, *Sm*-p80/GLA-SE has now been approved for phase I clinical trial (9).

As schistosomiasis candidate vaccines are progressing from phase I studies to testing in the target populations in several endemic settings, the distinctive disease exposure, co-infections and transmission settings that uniquely shape the immunological profiles may result in heterogeneity in vaccine responses. Hence, in addition to the existing logistical, accessibility and resource-limitation challenges, the immunological complexity presents exceptional challenges to vaccine development and testing in schistosomiasis endemic regions.

### Prior/Current Infection With Schistosomes and Antigen Sensitization

Generally, prophylactic vaccines are given to naïve individuals but for potential *Schistosoma* vaccines, this may be challenging as sensitization to *Schistosoma* antigens is likely to occur early in life or even *in utero* in endemic settings (40). Prior exposure to antigens included in a vaccine may enhance or suppress vaccine responses.

At birth, the offspring of *Schistosoma* infected mothers already show signs of B and T-cell sensitization to parasite antigens (41), with detectable schistosome-specific antibodies (including IgE), T cell responses in cord blood, and schistosome antigens in the urine of new-borns (40, 42). Even with zero egg counts, antibodies in infant serum recognize some worm antigens (43, 44). Following childhood exposure, infection peaks in 6–15 years old children (19) and can be as high as 90% in <12 years olds (45, 46). The peak and dispersion in worm burden gradually declines with age (46), which could be a reflection of reduced exposure or, in high exposure settings, the induction of naturally acquired immunity. Naturally acquired immunity is characterized by a broadening of antibody repertoire and a switch from predominantly egg-specific IgG1, IgG2, and IgM antibodies in infancy to a protective larval and adult worm-specific IgE antibodies toward adulthood and increasing duration of exposure (43, 47–49). IgE response, elicited after repeated exposure and or PZQ treatment is associated with protection (though not exclusively) in endemic populations (10). Natural or PZQ-driven death of adult worms releases parasite antigens from migrating larvae and this induces IgE response (50) which mediates antibody-dependent-cellular-cytoxicity (ADCC) killing of more parasites (31). Although not useful in vaccine development due to the potential to induce hypersensitivity (10), an IgE mediated protective immunity is subsequently developed in individuals, over many cycles of infection and or treatment (51).

This raises the question of how pre-exposure and pre-existing immunity influences vaccine responses. Depending on the vaccine antigen and the pathogen, the effect of pre-exposure may vary. For instance, reduced cytokine responses to Bacillus Calmette-Guérin (BCG) vaccination have been linked to previous exposure to other mycobacteria, but studies with hepatitis B vaccines show conflicting results with increased antibody responses after pre-exposure in one study, and lower in another study (52). For schistosome antigens, there is no evidence so far that pre-existing exposure to vaccine antigens negatively impacts schistosomiasis vaccine responses. In the r*Sh*28GST Phase 3 study, pre-existing immune responses to the 28GST antigen were observed in children aged 6–7, but there was no evidence of pre-existing anti-28GST responses negatively impacting vaccine immune responses (18). In addition, in studies with recombinant *Sm*14, high levels of the Th1 cytokines IFN-γ, and TNF-α could be observed despite a history of chronic exposure (53). This is in agreement with animal experiments which showed that successful immunization could be achieved in previously infected and cured baboons (39). As such, current data suggest that schistosome vaccines may retain immunogenicity despite prior exposure. However, the impact of prior exposure on vaccine responses may differ for each antigen and should therefore be carefully assessed in early-stage trials to avoid reduced efficacy results in phase three trials or implementation settings.

### Population Differences in Immune Profile Affecting Vaccine Responses

Differences in the baseline immune profiles between populations are known to affect both quantitative and qualitative response to vaccines (54). Since novel vaccine candidates are typically assessed for phase I safety trials in a non-endemic European population, it should be borne in mind that generally lower vaccine responses are found in African populations (55–58).

The immune profile of African cohorts, in contrast to Caucasian populations, typically contains more exhausted and activated NK cells, differentiated T and B cells and pro-inflammatory monocytes (56, 59). This immune profile was associated with a low vaccine-specific neutralizing antibody response and a poor efficacy to the yellow fever vaccine 17D (YF-17D) as compared to a Swiss cohort (56). BCG vaccination in African adults and children elicited low IFN-γ response and a mixed cytokine profile in a setting where BCG efficacy is low, but a predominant Th1 cytokine profile in the United Kingdom where BCG efficacy is 50–80% (55, 57, 60). Also, African infants did not show increase in the magnitude of T cell response to the MVA85A TB candidate vaccine unlike their UK counterparts (61). Furthermore, the HIV-adenovirus-vectored (Ad26.EnvA.01 and Ad35-EnvA) candidate vaccines induced better efficacy and greater T cell responses in an American cohort compared to South and East African subjects (58). These findings suggest that the baseline immune profile of African populations may interfere with adequate vaccine responses and this effect is especially important for the viral vectored and live vaccines such as the MVA85A and Ad26/35-EnvA, and BCG and YF-17D, respectively (55, 57, 61).

Genetic difference between populations could contribute to heterogeneity in vaccine responses. Vaccine immune responses are regulated by multiple gene complexes and networks which may be subject to genetic variations such as inherent cytokine and HLA gene polymorphisms (62, 63) and thus, difference in immune phenotype and functional response. For instance, Indonesians were shown to express the unique CD11c^+^ IL-10 producing B cell subset as compared to Europeans (64), depicting a possible genetic disparity. In a malaria endemic region in Mali, both adults and children exhibited an atypical FcRL4+ expressing memory B cell population unlike their American counterparts (65). Furthermore, volunteers of Congolese origin expressed a distinctly high number of *STAT6* and *IL10RA* regulatory gene polymorphisms, a genetic evidence that explains the predominantly asymptomatic-uncomplicated malaria infection manifestation in this population (66). Differences in response to vaccines such as the measles vaccine have also been attributed to genetic polymorphisms in the *CD46* and *SLAM* gene receptor (67).

However, there is increasing evidence suggesting that inter-population differences in immunological profiles are driven by non-inheritable environmental factors such as prior and current infection status which may typically be vastly different between rural vs. urban living (59, 68, 69). For example, rural African populations express a superior immune activated state, larger memory pool and Th2 polarization as compared to urban Africans (64) and Indonesians from a non-endemic urban setting showed a similar immune profile to European subjects yet markedly differed from Indonesians from an endemic rural area (59). More so, a type 2 immune profile comprising of Th2 cells, IL-4, IL-5, and IL-13 cytokines is typical of schistosomiasis high-exposure endemic populations (70). Subjects from the endemic setting exhibited a high frequency of CD161^+^Th2 cells, CCR6-KLGR1^+^ ILC2, CTLA^+^ T regs and IL-10^+^ CD11c^+^ B cells (64). Subjects from a rural setting in Uganda showed a decreased specific cytokine and antibody response to tetanus toxoid and *Mtb* purified derived protein (PPD) compared to their urban counterparts which persisted after adjusting for helminth infection (71).

Therefore, early phase clinical trials should preferably be conducted in target populations to ensure no gaps in translation at a later stage. In addition, booster regimens, potent adjuvants and higher antigen dose can be considered particularly for endemic populations such as in SSA where low vaccine response and efficacy is predominant.

### Co-infections

Besides prior and current schistosome exposure, schistosomiasis endemic populations are also highly diverse with regards to exposure to other infections. Chronic viral infections and poly-parasitism in schistosome endemic populations constantly expose the host immune system to a complex array of antigens and epitopes causing antigenic competition and immune sensitization (72). In addition, parasitic infections such as lymphatic filariasis, onchocerciasis and leishmaniasis often occur in schistosome endemic areas. This trains and pre-sets the immune phenotype and functionality, potentially distorting the host response to, vaccine candidates.

Noteworthy, in this review we focus on the concomitant parasitic (malaria and soil-transmitted helminths) and viral (Cytomegalovirus) infections which are highly prevalent among schistosomiasis endemic populations in the low and middle income countries (LMIC) (73, 74). Parasitic co-infections are of particular importance due to the significant negative impact on immunization especially among parasite endemic populations which are the target for schistosomiasis vaccine trials (73).

#### Malaria Co-infection

Malaria and schistosomiasis are both parasitic diseases that share co-endemicity, and cause significant morbidity and damaging socio-economic effects (75). With schistosomiasis only ranking second to malaria, these two parasitic diseases exhibit an overlapping geographical distribution pattern and a high prevalence of co-infection across the tropics and sub tropics (75). The prevalence of malaria is highest in children (76) and is mainly caused by *Plasmodium falciparum*. In fishing communities, which are a target population for schistosomiasis vaccine trials due to the high schistosomiasis transmission and intense exposure, the risk and prevalence of malaria co-infection is very high (77). The presence of breeding sites in the lake environment such as stagnant pools of water on the shores and unused old boats, fish bait mines and finger-ponds traps which sustain a heavy vector population in these communities (78). Also, fishing activities such as nocturnal fishing promote outdoor transmission as well as the temporary makeshift housing that is porous to the malaria vector (79).

Primary malaria infection induces CD4+ T cell differentiation into CXCR+ T follicular (T*fh*) cells to provide B cell help and the highly proliferative Vγ9+Vδ2+ T cells produce high levels of IFN-γ (80). Production of pro-inflammatory cytokines and chemokines such as IL-1β, IL-6, IL-8, IL-12, IFN-γ, and TNF induce fever and other signs and symptoms in previously unexposed individuals resulting in severe and fatal malaria (81). Increased TNF levels are associated with severe and cerebral malaria infection although sustained high levels result in reduction in parasitemia and improvement of fever (82, 83). However, this Plasmodium-induced inflammatory response also controls parasite replication and resolves infection through IFN-γ and TNF mediated killing of parasite (84). Meanwhile, acute uncomplicated infection activates the less functional CXCR3+PD1+ T*fh* cells which enhances production of IL-21 and pro-inflammatory cytokines (85). *P. falciparum* malaria infection additionally prompts dysregulation of B- and CD4+T cell function subsequently, causing significant and rapid loss of *Pf*-antigen-specific antibodies (85, 86). Asymptomatic malaria is an important consideration for clinical trials since it is the most common form among adults, the ideal early-phase trial subjects in endemic settings. Asymptomatic malaria is characterized by increased Treg numbers, IL-10 and TGF-β production, and dampened TNF, IFN-γ pro-inflammatory response characterizes (87). *P. falciparum*-induced upregulation of IL-10 production by CD4+Foxp3- Th1 cells rather than T regs, is usually sustained in persistent asymptomatic infection (88). Malaria infection additionally affects B cell phenotype and function. In repeated malaria infections and persistent asymptomatic parasitemia, accumulation of atypical memory B cells, a hyporesponsive anergic B cell subset expressing several inhibitory receptors, is seen (89, 90). Expansion of atypical memory B cells associated with clinical immunity following acute malaria has also been reported (91). This immunological memory may have implications on vaccine response given that it may distort the balance between antibody affinity maturation and B cell clonal selection following vaccination (90, 92).

Acute malaria has been reported to lower response to tetanus and diphtheria toxoids, meningococcal polysaccharide, *Salmonella* and *Haemophilus influenza* conjugate and whole vaccines (93–95). Fever associated with malaria infection contributes to a diminishing response to *Haemophilus influenza* vaccine (94). More so, asymptomatic parasitemia in malaria infection is associated with a decreased response to the newer acellular pertussis and meningococcal vaccine (85). Similarly, malaria infection during pregnancy and in infancy decreases antibody response to infant measles vaccination (96). These findings suggest and support treatment of malaria before vaccination is necessary to alleviate the antagonizing immune effects. Indeed, there is evidence that the response to some vaccines (such as the tetanus vaccine) improve following malaria chemoprophylaxis (93). However, pre-treatment does not improve response to or even impair immunogenicity of some vaccines such as the measles vaccine (93).

In co-endemic populations, it seems reasonable to test and treat malaria infections prior to vaccine administration. However, the added complexity of malaria pre-treatment in implementation settings makes it imperative to investigate the effect of asymptomatic malaria on vaccine response before phase 3 trials are performed. In addition, the optimal timing of malaria chemotherapy with regards to vaccine administration will need to be considered in order to optimize effects in different malaria transmission settings.

#### Soil-Transmitted Helminths (STHs) Co-infection

Soil-transmitted helminths (STHs) and schistosomiasis are predominantly co-endemic (97). It is estimated that one-third of the population in SSA is infected with one or more STH (98). This prevalence is driven by the overlapping poverty-related conditions of poor environmental hygiene, improper waste disposal, in adequate water supply and pollution of water bodies (99). The common STHs are the round worm (*Ascaris lumbricoides)*, hookworms (*Necator americanus* and *Anyclostoma duodenale*), whipworm (*Trichuris tichuria)*, and *Strongloides stercoralis* (97). However, hookworm and A. *lumbroicoides* infections seem the most prevalent of helminth co-infections in schistosomiasis infected individuals in many endemic areas (100, 101). Young children and males are more prone to the infections due to poor hand hygiene practices and active behavior that exposes them to contaminated soils and water (99). This high prevalence among adult males is an indication of a greater likelihood of existing or past infection, a caution for possible distortion of host immune response to other infections and vaccines. And also a challenge to the selection of trial subjects in co-endemic settings.

STH infection impairs development of protective immunity possibly as a result of the potent chronic suppression of the Th1 response required for protection against pathogens (72). STH infections typically induce a type-2 biased immune response (102). Therefore, elevated levels of IL-4, IL-5, IL-13, IgE in addition to general modulation of both innate and adaptive immune systems can be expected in STH co-infection (102). Chronic STH infection not only induces potent local but also systemic down regulation of the immune system. For instance, human subjects challenged with *N. americanus* exhibit a strong local and systemic Th2 and T reg response with high levels of IL-10 and TGF-β production (103). In chronic STH infection, it is the increased functional activity of the FOXP3^+^ Tregs which mediates immune suppression, rather than an increased frequency in chronically infected human hosts (104).

This potentially affects not only susceptibility and outcome of concurrent infections but response and efficacy of vaccine candidates (105). Studies in both human and mouse models report poor immunogenicity of the BCG vaccine in STH infected subjects (106). Decreased T cell proliferative response to BCG in helminth infected children was found to result from an enhanced T reg functionality and subsequent immune suppression during chronic infection (104). Besides, the immune profile to recombinant cholera toxin B subunit following live oral cholera CVD 103-HgR vaccination in helminth infected subjects is characterized by low IFN-γ, IL-2, and IL-12 (107), indicating a suppressed immune response. Particularly, existing *Onchocerca* infection significantly decreases immune response to tetanus vaccination compared to subjects without helminth infection (108). On the other hand, STH infections do not alter IgG antibody responses to previously administered measles and tetanus vaccines (109). Hence, with the changing lifestyle, better hygiene practices and anti-helminth treatment, there is a gradual reduction in helminth infections in many LMIC and one would expect better vaccine responses. This has indeed been shown in animal studies and in humans, findings from number of studies do support that treatment of STHs alleviate immune suppression and improve vaccine responses as reviewed elsewhere (110).

Helminth treatment, usually by oral administration of benzimidazoles i.e., albendazole (400 mg/kg) and mebendazole (500 mg), before vaccination may be beneficial for boosting vaccine responses. Deworming of subjects with Albendazole before BCG vaccination was reported to enhance BCG vaccine-specific response (111). A similar boosting effect on oral cholera vaccine among helminth infected subjects is exhibited (112). This is possibly due to alleviation of the helminth-induced immune suppression by anti-helminth treatment. Albendazole deworming is associated with enhanced pro-inflammatory responses and down regulation of inhibitory receptor expression CTLA, by the immune suppressive Tregs (113). However, one study did not find any significant effect of albendazole treatment on influenza vaccine response, despite the increased total IgA titres in the anti-helminth treated group (114).

Nevertheless, testing and treating of helminth infections may be beneficial in phase I schistosomiasis vaccine trials. Treatment before vaccination would alleviate the immune suppression induced by helminth infection prior to vaccine administration and thus, enable better response to vaccines. Meanwhile, treatment around time of vaccine administration possibly drives antigenic unmasking to expose more antigens for host immune recognition. However, antigenic unmasking, following PZQ treatment of intense pre-existing infection which results in release of worm antigens in excess of vaccine antigen, poses a risk of antigenic competition. This may result in poor and or non-specific immune response following PZQ treatment and vaccination. In this case, intensity of pre-existing infection should be taken into consideration. It should be noted that the duration and intensity of anti-helminth treatment itself does have a differential effect on B and T cell response (115). Therefore, not only the effect of STH infections but also their treatment on vaccine response and efficacy should be investigated in late-phase trials in endemic settings.

#### Human Cytomegalovirus (CMV) Co-infection

This double stranded DNA and enveloped virus belonging to the *Beta-herpesvirinae* infects between 40 and 100% of adults, varying in prevalence between populations (116). Approximately 90% of the infection incidence occurs in the LMIC of Africa, Asia and South America where up to 60% of the young adults and 90% of elderly adults are sero-positive (117–119).

Besides the high prevalence, the induced immune modulation and establishment of a periodically re-activated life-long latent infection marks CMV a very important viral infection (120, 121). CMV infection typically distorts the T cell repertoire and overall phenotype by inducing a rapid differentiation and clonal expansion of CMV-specific CD8+ T cells. This T cell subset can constitute up to 20% of the T cell repertoire, especially in older adults, but is not functional in controlling the infection (122). Meanwhile, the CMV-specific CD4+T cells increasingly produce IFN-γ, and MIP-1β that mediate re-activation of other latent infections in the host such as Tuberculosis (123). Chronic CMV infection thus causes a continuous accumulation of highly differentiated effector memory T cells. Besides, expression of co-stimulatory receptors in these T cells is inhibited, together resulting in a phenomenon known as “memory inflation” (124, 125), whilst at the same time depleting the naïve T cell pool (126).

Despite the universal host immune phenotype, the negative effect of CMV infection on vaccine responses is most pronounced in the elderly (120). CMV sero-positive elderly adults have been reported to exhibit a decreased response to *Influenza* vaccine (127, 128). A defective CD8+pSTAT1 and pSTAT3 pathway, expanding senescent CD57+KLG1+ T cell repertoire and the increasing TNF-α and IL-6 levels with infection duration, reduces cytokine responsiveness of vaccine-induced T-cells (120, 128, 129). Findings in younger CMV infected adults are conflicting. An enhanced response, characterized by high levels of circulating Th1 and Th2 cytokines to *Influenza* vaccine is seen (127), possibly due to the cross-reactive CMV-specific CD8+ T-cell epitopes (130). Meanwhile, response to Ebola candidate vaccines, ChAd3 and MVA, is impaired (120).

Given that CMV establishes a life-long infection that cannot be cleared or treated, the attention for vaccine trial design and implementation should rather be focused around different age of target populations. Phase I trials may consider testing and exclusion of CMV sero-positive subjects, especially in CMV low and moderate prevalence areas, for any vaccine targeting young children or infants. However, this may not be possible in populations where CMV infection is prevalent and almost universal, even among children. For late-phase trials, inclusion of CMV sero-negative vs. sero-positive subjects, could help decipher possible interaction between CMV and vaccine responses. Alternatively, general understanding of the possible effect of CMV could first be investigated through fundamental studies and then regimens such as booster doses for the sero-positive subjects in late phase trials can be considered and tailored accordingly.

#### Other Co-infections

Prevalent co-infections with obvious immune-modulation and even immune incompetence induction are HIV and TB.

HIV/AIDS, a life-long viral infection with no cure presently and the cause of profound immune-incompetence, is a prevalent viral infection in SSA and other developing regions of the world (131). Over 20 million people are infected in South and Eastern Africa alone (132). HIV establishes a preferential infection and depletion of CD4+ T cells, increases T cell activation, exhaustion and death, impairs antigen presentation, CD4+ and CD8+ T cell functionality (131, 133). This results in very low CD4+ T cell count (below the 500 cells/mm^3^ lower limit), severe immune impairment, poor vaccine response as well as rapid vaccine induced decline (133, 134).

Combination anti-retroviral therapy facilitates immune reconstitution and viral load suppression, despite persistence of the virus in latent reservoirs (133), suggesting a near-optimal immune response can be expected. Indeed improved vaccine response and reduced risk of infection following Influenza, PPV23 and PCV pneumococcal vaccinations has been reported in patients on cART (135–137). Nevertheless, immune responses in HIV infected patients may be lower, or wane off quicker, despite cART therapy as has been demonstrated in trials with hepatitis B, Influenza, experimental TB and BCG vaccination both in adults and children (138–143).

Despite the potential public health importance of vaccination in HIV infected patients given their increased susceptibility to vaccine-preventable diseases (144), they are often excluded from vaccine trials because of safety concerns and unfavorable benefit/risk profiles (145, 146). For example, the unfavorable benefit/risk profile of BCG vaccination in HIV infected has to led its constant contraindication (146–148). Therefore, testing for HIV, exclusion of sero-positives from trials and referral for cART treatment, is paramount for safety. However, given the high percentage of HIV positive individuals in some areas and the increased use of cART and non-viable vaccines, these populations should be included at a later stage clinical testing of the vaccine to expedite roll-out in these populations after potential registration.

Tuberculosis is another prevalent co-infection among schistosomiasis endemic populations in LMICs affecting over 10 million people and causing significant immune-modulatory impact on infected hosts (132). In active TB infection, levels of IFN-γ, IL-1β, IL-18, are severely decreased and Treg numbers rise thus immune suppression ensues in the infected host. Consequently, a high FOXP3, TGF-β, and IL-4 and low IFN-γ, expression in active TB patients is associated with poor response to BCG vaccination (149, 150). However, an increased antibody response to several unrelated antigens such as measles and tetanus toxoid in active TB patients has also been reported (151).

Similar to HIV infection, active TB is a factor for vaccination deferral due to safety reasons. Nevertheless, further studies within and or outside vaccine trial studies may be important to ensure no interaction occurs between latent TB infection and schistosomiasis candidate vaccine response and efficacy.

The current COVID-19 pandemic has led to over 1.9 million deaths globally (152) inducing serious, potentially long-term immunological perturbations in the infected host that may influence vaccine responses in the same individual. The severity of the COVID-19 disease is associated with the hyper-inflammatory response, “cytokine storm,” especially in the presence of co-morbidities such as diabetes and obesity which are characterized by elevated levels of pro-inflammatory IFN-y,IL-1B,IL-12,IL-6,IL-27, and TNF cytokines (153). It has been suggested that the Th2 biased, T-reg, IL-10 mediated immune hyporesponsive and controlled inflammatory state in schistosomiasis infection (154) could counteract the damaging effects of COVID-19 disease and limit morbi-mortality (155). This theory could explain the relatively low severity and fatality rates of the COVID-19 in the helminth-endemic Africa (156, 157). However, there is currently no evidence to prove this COVID-19 and helminths interaction.

#### PZQ Co-administration

**Figure 1:**
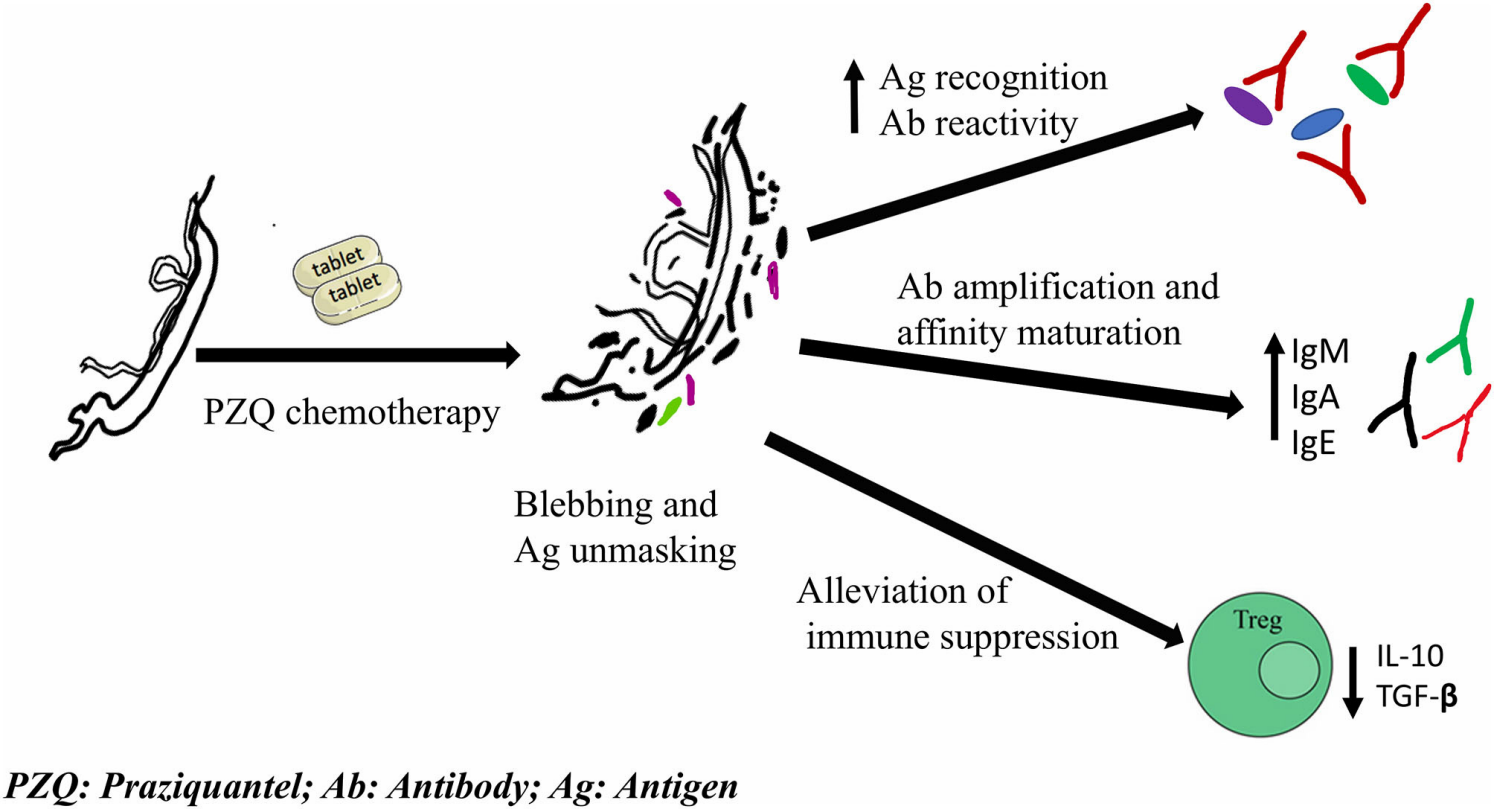
Schematic summary of PZQ-induced immune-modulatory responses that potentially impact vaccine response upon co-administration/delivery. PZQ acts on parasite tegument causing vacuolation and blebbing. This unmasks worm antigens and elicits an increased antigen recognition, antibody reactivity, amplification and affinity maturation, and alleviates the immune suppression induced by live parasites.

PZQ, an acylated isoquionoline-pyrazine derivative, is the main drug used in the treatment of schistosomiasis and the basis of community-based mass drug administration (MDA) programs for the last 30 years (51, 158). PZQ, administered orally at dosages of 40–60 mg.kg^−1^, is efficacious against adult worms with low toxicity and safe, even in pre-school age children and in third trimester pregnancy (159, 160). Although the mechanism of action of PZQ is not entirely understood, it is widely accepted that it disrupts the worm calcium homeostasis leading to worm muscle contraction and paralysis (158, 161, 162). The ensuing PZQ-induced tegument vacuolation and blebbing exposes the worm surface antigens, previously concealed in the intact live parasite to host's immune system (Figure 1) (162). *In vivo* studies show extensive structural changes in the tegument, sub-tegumental and gastro-dermal musculature, causing leakage of tegumental cytoplasm from live worm in the first 15 min of PZQ treatment (163). Rodent studies have shown that PZQ-induced tegument disruption leads to exposure of a previously concealed native GST enzyme 90 minutes post PZQ treatment (164). Also in humans, drastic increases in cytokine release after PZQ treatment suggests that chemotherapy results into a pulse release of antigens (165) (Figure 1). This suggests that PZQ not only exposes tegument antigens but also cytosolic antigens and enzymes essential for parasite survival to the host immune system, enhancing further tegument damage and parasite killing.

As a consequence of antigenic unmasking, PZQ treatment may enhance antigen recognition of antigen-protein-isoforms that would otherwise be hidden (166). Post-treatment serum samples from *S. haematobium* patients have been shown to recognize a greater number of proteins compared to pre-treatment sera. More so, some proteins and their isoforms are recognized only in post-treatment serum samples (166). Noteworthy, the proteins that show enhanced recognition post treatment are generally those that are associated with parasite musculature and glycolytic metabolism (167), consistent with the disruptive mechanism of action of PZQ. This enhanced antigen recognition permits better neutralization by antibodies raised by vaccination (164, 168). Increased antigen recognition consequently enhances affinity maturation of immunoglobulin responses such as worm specific IgE. For instance, high IgE titres following PZQ chemotherapy ensues from directed IgG affinity maturation induced by PZQ (167), an effect which may also be conferred to co-administered vaccines and moreover lead to a more sustained antibody response following vaccination (164). Besides, antibody titres IgG1, IgG2, IgG4, IgM against worm antigens and IgG2, IgE to egg antigens are boosted following PZQ administration (169).

On the other hand, antigenic competition as a result of PZQ-induced parasite killing could suppress response to the single vaccine antigens. This is because PZQ-induced parasite killing avails large amounts and or a variety of crude worm antigens into the blood stream, in excess of vaccine antigen load. This can potentially overshadow vaccine response or elicit non-specific or undesirable immune response (170). In the phase 3 human trials of the r*Sh*28GST vaccine candidate, PZQ was administered before and after the initial vaccination as well as just before and after the booster vaccination. No significant delay in schistosomiasis recurrence was seen in the vaccine group compared to the control group despite vaccine induced immunity (18). Although this may be attributed mainly to the failure to induce the desired IgG3 and IgA protective antibody response but rather IgG4 responses, interference by the repeated PZQ treatment was also suggested to have, partly, contributed to the observed results (18). Previous studies have demonstrated PZQ-induced modulation of cellular cytokine response to GST (23, 24). Likewise, more subjects exhibit an increased Sh28GST specific cytokine with a shift toward a more pro-inflammatory phenotype following PZQ treatment than before treatment (23, 24). However, a definite conclusion on the role of the PZQ treatment in the outcome of this trial cannot be made. Preferably, next studies should include a control arm whereby the timing of PZQ treatment is shifted several weeks before or after the vaccine administration.

Indeed, PZQ treatment markedly alters both the polarization and magnitude of schistosome specific cytokine responses with longer-term immunological impact than just transient clearance of infection. In the human host, Th2 biased response in form of increased parasite-specific IgE, eosinophil numbers, soluble-high and low affinity IgE receptors on eosinophils and CD23+B cells following PZQ administration is observed (23, 171, 172), which has been related to resistance to reinfection (172–174). In addition, PZQ chemotherapy alleviates the immune suppression induced by schistosomes by downregulating the immune dampening T-reg, Th17 cell numbers and IL-10 cytokine levels (23, 24, 175, 176). Clearance of infection following PZQ administration results in increased effector T cell frequency, increased Schistosome-specific cytokine response and decreased levels of T reg. This contrasts with the immune suppressive profile during infection such as antigen specific hypo-responsiveness, T cell memory pool distortion and increased CD4+CD25+FOXP3+T cell numbers (177). Consequently, in a randomized trial in Entebbe, Uganda, PZQ treatment significantly, although transiently, improved vaccine response to measles immunization in *S. mansoni* infected pre-school children (169). Noteworthy, this effect seems greatest when PZQ is administered a few weeks before immunization (169).

In conclusion, although PZQ has been deemed to be non-immunogenic in itself and thus have no impact on vaccine efficacy, PZQ-induced antigenic release elicits both transient and long term immune modulatory effects (23, 165, 175, 178) (Figure 1) which in turn, potentially augment or mask vaccine responses. This is particularly important given that future schistosome vaccine trials in endemic settings target adult subjects, whose immunological and exposure profile has been shaped by repeated exposures and or PZQ treatment. As such PZQ-vaccine co-delivery could either be a very effective, more wholistic strategy to enhance vaccine response and efficacy or be detrimental to detecting vaccine efficacy in late-stage clinical trials. Therefore, different vaccine schedules with PZQ chemotherapy needs to be assessed in order to select the most appropriate vaccine-PZQ combination and immunization-chemotherapy treatment schedule before embarking on such strategies in phase 3 studies.

## Outlook

Given the potentially complex interaction of the above mentioned factors within the target population (43), the appropriate selection of trial subjects in which confounders have been taken into account appropriately, has become a true challenge to vaccine development and testing. Recognizing the fact that large phase 3 efficacy trials will be performed in a randomized fashion, most likely correcting for potential unknown or unidentified confounders within the trial, understanding or recognizing the potential of these factors will ultimately be important for extrapolation of trial results and implementation of any schistosome vaccine across sub-Sahara Africa. Moreover, the phase 3 design will have to take into account the treatment of co-infections in the context of already existing MDA programmes.

Therefore, we argue that considerable investment should be made during early phase clinical testing in an attempt to understand and disentangle these confounders. As a start, thorough screening of subjects for co-infections, chronic diseases and physiological disorders should be done in order to identify confounders in early-stage clinical trials, and enable appropriate selection of trial populations with sufficient heterogeneity at a later stage. The inclusion of several trial arms with different population subsets (e.g., high vs. low pre-exposure) is helpful to unravel the interaction between co-infections and vaccine responses. For treatable co-infections, the effects of pre-treatment such as PZQ, artemisinin and or albendazole administration at the same time or prior to vaccination should be carefully incorporated into trial designs especially from phase II trial stage. Additionally, timing of treatment and vaccine administration maybe a crucial determining factor for PZQ masking of or synergy with vaccine response and efficacy. Understanding the interplay between drug administration and vaccine responses will prove to be essential for implementation of novel vaccines in MDA settings. In the case of co-infections such as HIV/AIDS (which can be treated but not eliminated), testing and exclusion of sero-positive volunteers is the current practice in phase 1 trials. However, including seropositive individuals in phase II and III trials should be promoted to ensure population-wide implementation of the vaccine after registration.

In larger, late-stage stage clinical trials, the predominantly age dependent distribution of infection intensity and development of protective immunity among endemic populations (46) supports the need for careful selection of analysis methods and tools for handling trial data. Implementing approaches such as pre-defined sub-group analysis, hierarchical clustering analysis and stratified randomization with regards to age, and minimal and intense prior exposure in trials is likely to be useful. This would help to explicitly identify the immunologically distinct heterogenous groups and give useful insights into differential vaccine efficacy.

Controlled Human infection (CHI) models present an opportunity to not only disentangle the different parameters at play in endemic settings, but also accelerate the vaccine development pipeline in general. In the implementation of the CHI model, healthy volunteers are intentionally infected with a pathogen with the aim of generating knowledge on natural history of a disease, testing vaccines or therapeutics, and developing reliable and defined models of infection for future studies (179). These studies are controlled in terms of dose and route, production and selection of the pathogen strain administered. Additionally, a “controlled” condition/environment is key to prevent natural infection during the study period (179). Therefore, signs and symptoms of disease, and evolution of responses following a defined timing of the exposure can be observed in a well described and managed manner. This makes it possible to study the natural history of infection, host-pathogen interactions, evolution of immune responses and provide a preliminary assessment of vaccine efficacy (180, 181). The CHI models are also proof of concept studies and furthermore enable identification of correlates of protection. This approach allows for testing and early selection of promising vaccine candidates in a smaller group of healthy volunteers (<100), within a shorter period of time under controlled conditions compared to the classical vaccine testing methods (182). Hence, CHI studies reduce the burdening cost and time requirements, and the high downstream vaccine efficacy failure, associated with classical clinical trials approach. Noteworthy, for safety and ethical concerns, the CHI approach is applicable only for infections/diseases that are self-resolving and or are treatable such as schistosomiasis (179, 180).

The recent development of a controlled Human Infection model for Schistosomiasis (CHI-S), utilized single-sex cercariae for infection of healthy Dutch volunteers (181). This approach prevents the risk of egg-related pathologies as seen in the “natural” mixed-sex schistosome infections, while preserving the ability to cause infection and mature into adult schistosomes in human subjects (183). The highly sensitive diagnostic test, Circulating Anodic Antigen (CAA) test is used to detect infection that usually occurs between 6 and 12 weeks after the CHI procedure (181). Subjects are then treated with PZQ at 8 or 12 weeks post exposure to ensure clearance of infection (181). The first CHI-S trial demonstrates that the male-only CHI-S is safe and tolerable among naïve Dutch volunteers with about 82% infection rate, and absence of schistosome egg production (181). Despite obvious limitations of the single-sex nature of the CHI-S model, it can nevertheless be used as a screening tool for future schistosomiasis vaccine candidates and new drugs. More importantly, the implementation of the CHI-S model in endemic settings provides a unique opportunity to disentangle the aforementioned distinctive immune responses and potential natural resistance, to Schistosoma infection and vaccines among endemic populations (184). This would not only accelerate vaccine development and testing but also help unravel the heterogeneity in vaccine immunogenicity, safety, and efficacy.

## Conclusion

The urgent need for a vaccine to eliminate the burden of the schistosomiasis in LMICs calls for acceleration in the testing and subsequent approval of the current vaccine candidates. However, endemic populations exhibit a multiplicity of distinctive disease transmission, exposure, pathogenesis, genetic and environmental factors that result in immunological challenges as summarized in Table 1. These potentially impact vaccine responses and efficacy and amplify the chances of vaccine failures. Therefore, significant consideration has to be conceded to these immunological challenges in order to accelerate vaccine development and testing. Studies to investigate and accurately establish the impact and extent to which these immunological challenges affect host immune and vaccine response should become a fundamental facet of baseline and or preparatory studies as well the actual trial protocols for vaccine testing in endemic settings.

**Table 1:** Summary of the immunological considerations for schistosome vaccine development.

**1. Prior/Current schistosome exposure**	**Immune-Modulation**	**Key references**	**Vaccine response**	**Key references**
a) *In utero* exposure b) Early childhood exposure c) Age-linked cumulative exposure	B and T cell sensitization Specific cord blood IgG, IgE ↑ IgG2 and IgM ↓ IgE ↑ Adult worm specific-IgE, IgM ↑ Antigen recognition ↓ Egg-specific IgG, IgG2 Age-dependent acquired immunity	(40) (41) (42) (185) (43) (167)	↓ BCG, *Pf* Sporozoite Vaccine No effect on r28GST response ↑ r*Sm*14 vaccine	(52) (186) (187) (18) (53)
2. Difference in baseline immune-profile/micro-environment	Activated immune micro-environment in African cohort ↑ Exhausted/activated NK cells ↑ Differentiated B and T cells Pro-inflammatory monocytes ↑ Genetic differences; cytokine and HLA gene polymorphisms CD11c^+^ IL-10+ B cell expression in Indonesians Atypical FcRL4+ memory B cell in Malian subjects High *STAT6* and *IL10RA* gene polymorphism among Congolese subjects ↑ Activated, memory pool Th2 polarization in rural populations	(56) (64) (65) (66) (59) (64)	↓ Yellow fever, HIV, BCG, MVA85A TB vaccines in African cohorts ↓ HIV Ad26.EnvA.01 and Ad35-EnvA candidate vaccines in African cohort Measles vaccine heterogeneity due to *CD46* and *SLAM* gene polymorphism ↓ Tetanus toxoid, Mtb PPD in rural subjects	(56) (55) (57) (58) (61) (58) (67) (71)
3. Co-infections a) Malaria	Primary infection malaria ↑ CXCR+ T follicular (T*fh*) ↑ IFN-γ producing Vγ9+Vδ2+ T cells Previously unexposed persons ↑ IL-1β, IL-6, IL-8, IL-12, IFN-γ, TNF Asymptomatic malaria ↑ IL-10, TGF-β, CD4+Foxp3- Th1 cells, Tregs ↑ TNF, IFN-γ Repeated and persistent infection ↑ Atypical memory B cells	(80) (81) (88) (89)	↓ Tetanus-diphtheria, meningococcal, salmonella, *Haemophilus influenza*, pertussis and meningococcal vaccines ↑ Measles vaccine upon malaria treatment ↑ Malaria RTS,S and r*Pf* circumsporozoite candidate Vaccine	(93) (94) (95) (84)
b) Cyto- megalovirus(CMV)	↑ CMV-specific CD8+ T cells ↑ IFN-γ and MIP-1β production Memory “Inflation” ↓ T cell co-stimulatory receptor expression ↑ Senescent CD57+KLG1+ T cells	(122) (123) (125)	↓*Influenza* vaccine in older adults ↑*Influenza* vaccine in young adults ↓ Ebola candidate vaccines, ChAd3 and MVA	(127) (128) (120)
c) Soil trans-mitted Helminth (STHs)	↓ Th1 response ↑ IL-4, IL-5, IL-13, IgE Treg, IL-10, and TGF- β	(102) (105) ↑ (103)	↓ BCG, Cholera, Tetanus vaccines No effect on previous measles and tetanus vaccinations	(104) (106) (107, 108) (109)
d) Human Immuno-deficiency Virus (HIV)	↑ T cell activation, exhaustion and death Antigen presentation ↓ T cell number and function ↓ Antibody decay	(133) (131) ↑ (134)	↓ Hepatitis B, Influenza, measles vaccines ↑ Influenza, PPV23 and PCV pneumococcal vaccine following cART	(139, 140) (135, 141, 142) (136, 137)
e) Tuberculosis (TB)	↓ IFN-y, IL-1β, IL-18 in Active TB ↑ T reg in Active TB ↑ Th1 in prior BCG vaccination	(149) (150) (188)	↑ Tetanus toxoid, other antigens	(151)
4. PZQ co-administration	↑ Antigen unmasking, recognition and Antibody neutralization ↑ Antibody maturation, isotype switching and Affinity maturation ↑ Specific IgE, IgA, IgG4, IgG2, and eosinophils Effector T cell frequency ↑ Th2 IFN-y, IL-5, IL-4, and IL-13 ↓ Tregs, Th17, IL-10	(166) (165) (171) (172) (174) (177)	↑ Sm28GST and measles vaccine, ↓ rSh28GST in phase 3 trials	(24) (169) (18)

*↑, increased and ↓, decreased*.

## Author Contributions

MR, AME, JPRK, and ED: conceptualization. ED and JPRK: writing (original draft preparation). MR, AME, AAS, MY, and SC: writing (review and editing). MR and AME: supervision. All authors contributed to the article and approved the submitted version.

## Conflict of Interest

The authors declare that the research was conducted in the absence of any commercial or financial relationships that could be construed as a potential conflict of interest.
